# Alteration of Skin Properties with Autologous Dermal Fibroblasts

**DOI:** 10.3390/ijms15058407

**Published:** 2014-05-13

**Authors:** Rajesh L. Thangapazham, Thomas N. Darling, Jon Meyerle

**Affiliations:** Department of Dermatology, Uniformed Services University of the Health Sciences, Bethesda, MD 20851, USA; E-Mails: thomas.darling@usuhs.edu (T.N.D.); jon.meyerle@usuhs.edu (J.M.)

**Keywords:** dermal fibroblast, cellular therapy, skin regeneration, wound healing, regional identity, volar, non-volar, dermal sheath, dermal papilla, hair follicle, hair follicle neogenesis, acral skin

## Abstract

Dermal fibroblasts are mesenchymal cells found between the skin epidermis and subcutaneous tissue. They are primarily responsible for synthesizing collagen and glycosaminoglycans; components of extracellular matrix supporting the structural integrity of the skin. Dermal fibroblasts play a pivotal role in cutaneous wound healing and skin repair. Preclinical studies suggest wider applications of dermal fibroblasts ranging from skin based indications to non-skin tissue regeneration in tendon repair. One clinical application for autologous dermal fibroblasts has been approved by the Food and Drug Administration (FDA) while others are in preclinical development or various stages of regulatory approval. In this context, we outline the role of fibroblasts in wound healing and discuss recent advances and the current development pipeline for cellular therapies using autologous dermal fibroblasts. The microanatomic and phenotypic differences of fibroblasts occupying particular locations within the skin are reviewed, emphasizing the therapeutic relevance of attributes exhibited by subpopulations of fibroblasts. Special focus is provided to fibroblast characteristics that define regional differences in skin, including the thick and hairless skin of the palms and soles as compared to hair-bearing skin. This regional specificity and functional identity of fibroblasts provides another platform for developing regional skin applications such as the induction of hair follicles in bald scalp or alteration of the phenotype of stump skin in amputees to better support their prosthetic devices.

## 1. Introduction

Faulty healing of cutaneous wounds impairs skin function and appearance due to scar formation, ulceration that predisposes to secondary infection, pain, or physical alterations that interfere with normal barrier function. Standard approaches to improve wound healing may speed reepithelialization but skin function is often compromised. A new approach to promote normal healing and skin regeneration is to alter the wound environment by introducing human cells into the wound or wound margin.

A major cellular constituent of the dermal layer of skin is the fibroblast. Fibroblasts are resident mesenchymal cells in the dermis that produce collagen and other extracellular matrix proteins. Fibroblasts are known to have a critical role in skin structure and integrity, and cultured autologous dermal fibroblasts are believed to promote skin regeneration and rejuvenation [1]. While there have been advances in the use of other cell types for skin regeneration such as keratinocytes [2], endothelial cells [3], bone-marrow derived mesenchymal cells [4], induced pluripotent stem cells [5], genetically modified fibroblasts [6], allogeneic cells [7], and skin substitutes [8,9], the focus of this review is the use of autologous fibroblasts in skin directed therapies.

The term fibroblast encompasses cells with variable attributes found in skin and other organs. The skin alone has many subtypes with unique properties. Increasing understanding of different fibroblast sub-populations has been exploited in the development of new therapies designed for skin rejuvenation via collagen production. Additionally, there is emerging research to suggest that fibroblasts have the ability to “reprogram” the skin for the purpose of skin rejuvenation in aging skin.

The first part of this review summarizes the roles of fibroblasts in normal wound healing and describes the therapeutic uses of fibroblasts in skin regeneration. In the second part, we explore regional and microanatomic properties of fibroblasts for altering skin phenotype.

## 2. Autologous Fibroblasts Promote Wound Healing

Wound healing involves four phases: hemostasis, inflammation, proliferation and remodeling [10]. Fibroblasts are critical during the proliferative phase of wound healing, which begins a few days after wounding, when fibroblasts begin to proliferate and migrate into the wound bed to produce extracellular matrix (ECM) proteins. ECM proteins act as a scaffold for inflammatory cell migration and granulation tissue generation. Granulation tissue provides the temporary substrate upon which re-epithelialization by keratinocytes takes place. During proliferation, fibroblasts also have the capacity to further differentiate into myofibroblasts that generate wound contracture. Later in the remodeling phase of wound healing, myofibroblasts undergo apoptosis resulting in a decreased cellular density while the remaining dermal fibroblasts begin to produce type I collagen. This plasticity of the fibroblast as it transitions from producing ECM to promoting wound contracture to the synthesis of Type 1 collagen makes it an attractive candidate for cellular based therapies in wound healing.

The plasticity of fibroblasts during wound healing implies that the regulation of fibroblast phenotype is complex. For example, the differentiation of fibroblasts into myofibroblasts is regulated by members of the TGF-β family that are released early in wounding by platelets [11]. Myofibroblast differentiation is also stimulated by platelet derived growth factor-CC (PDGF-CC) produced by M2 macrophages [12] and by Interleukin-22 (IL-22) produced by adaptive and innate lymphoid cells [13]. In addition to the production of ECM, fibroblasts secrete a variety of growth factors and proteases that promote or regulate wound healing [10]. For example, soluble factors such as fibroblast growth factor 2 (FGF2, also known as basic FGF), vascular endothelial growth factor (VEGF), and hepatocyte growth factor (HGF) promote wound healing. FGF2 stimulates wound healing and regulates ECM production and degradation [14], while VEGF stimulates angiogenesis [15,16]. Dermal fibroblasts in a fibrin matrix exposed to thrombospondin-1 produce increased amounts of VEGF that stimulates endothelial cell tubulogenesis [17]. Hepatocyte growth factor (HGF) has a variety of effects on wound healing, including stimulation of angiogenesis, regulation of matrix deposition and degradation, and stimulation of keratinocyte migration and proliferation [18]. The production of HGF involves intracellular signaling by molecules such as c-JUN [19] and integrin-linked kinase (ILK) [20], demonstrating that HGF production is secondary to growth factor stimulation and cell-matrix interactions. This complex web of interactions in the wound bed relies heavily on the role of fibroblasts as mediators of wound healing.

In general, wound healing is a well synchronized event; however, pathologic states are not uncommon. For example, fibroblasts from hypertrophic scars release increased amounts of TGF-β1 [21]. In contrast, fetal fibroblasts associated with scarless healing produce TGF-β3 [22]. The differential expression of TGF-β1 *vs*. TGF-β3 in hypertrophic *vs*. normal scars represents another target for cellular based fibroblast therapies.

Along the lines of differential expression of proteins by fibroblasts, specific proteases produced by fibroblasts, such as matrix metalloproteinase 2 (MMP-2), are involved during the remodeling phase of wound healing [23,24]. Fibroblast expression of MMP-9 and MMP-13 in the late remodeling phase is believed to promote scar-free healing [25]. This differential expression of favorable proteases represents another advantage of fibroblast therapies.

## 3. Autologous Fibroblasts for Cellular Therapies

### 3.1. Preclinical Studies and Approach

The realization that dermal fibroblasts have regenerative potential in skin repair and rejuvenation [26–29] has led to the development of cell therapies for a variety of skin indications. These include the treatment of surgical and burn wounds; chronic wounds such as diabetic and pressure ulcers; cosmetic indications such as treating facial rhytides; and hair growth in androgenetic alopecia. Toxicology studies of dermal fibroblasts have shown that they do not induce tumors or show oncogenic transformation when injected into mice [30]. In addition to this safety profile, there are several animal models in pig and rodent for preclinical evaluation of dermal fibroblasts in the developmental pipeline to bring the dermal fibroblast products to market.

In wound healing, a positive correlation between complete wound healing and fibroblast numbers in a porcine dermal substitute used to repair a full thickness skin defect showed the importance of fibroblasts in wound healing [31]. Additionally, autologous dermal fibroblasts from pigs were highly effective when transplanted to full thickness wounds in porcine wound models as demonstrated by accelerated re-epithelialization and enhanced survival of transplanted keratinocytes [32]. Not surprisingly, transplanted autologous dermal fibroblasts had better viability and resulted in improved re-epithelialization of wounds and accelerated wound healing when compared with allogenic dermal fibroblasts [33]. This is likely due to greater inflammation induced by allogeneic dermal fibroblasts, with a significant increase in scar formation and increased wound contraction, than autologous dermal fibroblasts, which induced normal restoration of cutaneous function with minimal scar [34]. Autologous dermal fibroblasts also resulted in improved healing in irradiated wounds, increasing the rate of wound healing, wound tensile strength, and resulted in higher cell densities compared to controls [35].

Dermal fibroblasts are also being evaluated for regeneration in non-skin tissues. For example, dermal fibroblasts combined with Bmp2 in a gelatin scaffold applied to full-thickness parietal defects produced *de novo* cranial suture regeneration after four weeks of engraftment. This technique has promise as a therapy for patients with congenital disorders of premature ossification of cranial sutures [35]. Dermal fibroblasts have been evaluated for other applications like tendon regeneration [36], closure of pleural defects for sealing airways [37], and for anterior cruciate ligament engineering [38].

### 3.2. Fibroblast Expansion and Production

Skin is an excellent source of fibroblasts and they are easily harvested by a punch biopsy of the normal skin. They can also be obtained from viable skin of debrided burn tissue [39]. Recent work highlighting the differences in gene expression and function of fibroblast from different anatomical sites [40,41] supports the concept that dermal fibroblasts should be selected from anatomic sites appropriate for the therapeutic indication.

**Figure 1.**
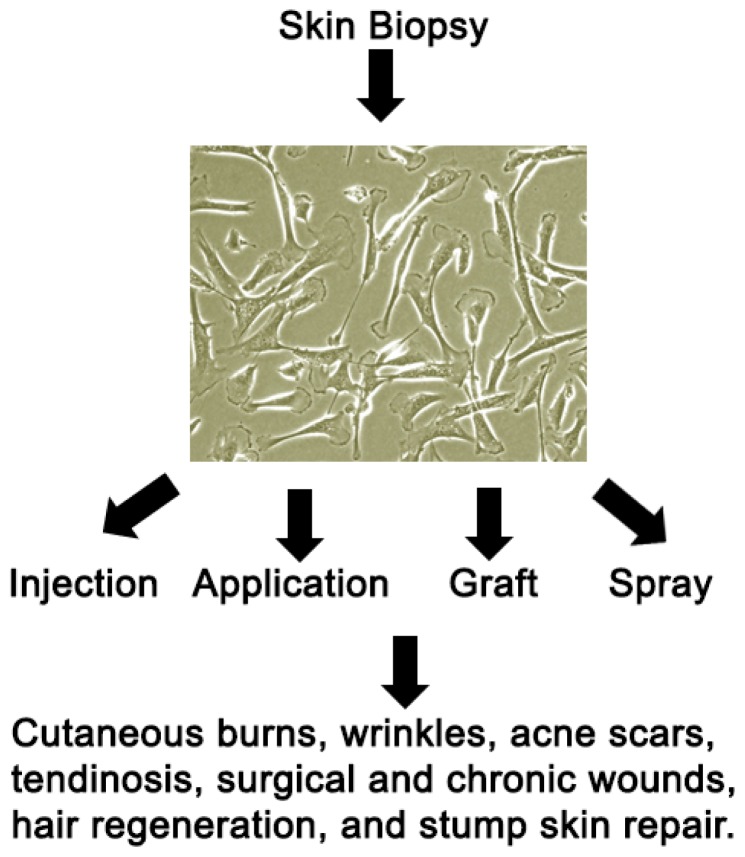
Flow diagram of harvesting skin, growing fibroblasts, and injecting for different uses. The location of the skin biopsy for growing fibroblasts may be directed by the therapeutic indication.

The production of dermal fibroblasts for therapy begins with a trained professional obtaining a skin biopsy. The biopsy is then sent to a Good Manufacturing Process (GMP) cell culture processing unit for dermal fibroblasts isolation by enzymatic methods, culture, expansion and purification (Figure 1). The cells are often resuspended in freezing media for cryopreservation or sent to the clinical facility for application. Advancements in cell culture techniques [42] to expand the cells without losing the unique cellular identity and regenerative capacity of the cells, is important for successful therapeutic application. Given that cellular expansion is expensive and time consuming, conventional flask based systems for tissue culture are being replaced by bioreactors and other novel methods to speed expansion and decrease manufacturing time.

While cellular therapy with autologous dermal fibroblasts has immense potential, many parts of the supply chain require development (Figure 1). These include advancements in isolating and culturing contamination free homogenous fibroblast populations, technology to rapidly grow optimal number of cells with desired potency, optimal harvest site selection based on desired therapeutic indication, and the storage and transport of the fibroblasts to the clinical site for application.

### 3.3. Mode of Administration and Cell Delivery

The US Food and Drug Administration (FDA) defines autologous use as “the implantation, transplantation, infusion, or transfer of human cells or tissue back into the individual from whom the cells or tissue were recovered” (21CFR 1271.1). The route of administration, mode of delivery, and the formulation of autologous dermal fibroblasts are determined by the indication. In general, injection is the most common delivery method. Direct injection involves fibroblasts being introduced into the dermis of the skin with a 27 or 30 gauge needle. In 2011 the FDA approved the use of autologous fibroblasts injected into the dermis to improve the appearance of moderate to severe facial nasolabial fold wrinkles in adults (LAVIV, Fibrocell Technologies, Exton, PA, USA). The treatment regimen for Laviv is the injection of 1.5 million autologous fibroblasts in 0.1 milliliter per linear centimeter into dermis in three treatment sessions at 3–6 weeks intervals [43]. Replicel Life Sciences Incorporated (Vancouver, BC, Canada) is developing a dermatology injector device to deliver cells in a nominal injection volume with minimal pressure simultaneously controlling needle depth and angle for its hair regeneration and tendon repair applications. Another delivery method is direct application of fibroblasts suspended in matrix to the wound or ulcer site. In diabetic foot ulcers a proprietary mixture of cultured keratinocytes and fibroblasts are suspended in fibrin glue and applied to the wound before the area is covered with a dressing [44]. Another approach is grafting a composite bandage with cells incorporated in a matrix. Several approaches have been developed for incorporating cells in a matrix. Fibroblasts are grafted either as a dermal equivalent (matrix and fibroblast) or bi-layered skin substitutes (dermal equivalent overlaid with autologous keratinocytes) at the wound site. Boyce and colleagues successfully developed a cultured skin substitute with autologous fibroblasts and keratinocytes that was grafted to the burn area [45]. This technique used collagen-glycosaminoglycan substrates as the matrix, while other investigators have used other matrices, such as a hyaluronic acid scaffold in an autologous bioengineered skin equivalent that was used to repair a large wound in a nine-year-old girl with after removal of a congenital giant nevus [46]. More recently, a spray-on system to deliver autologous cells directly to the wounded area has been developed. This system does not rely on expansion of the cells *in vitro* since the skin is harvested and digested enzymatically before application [47]. The spray-on system is being developed by Avita Medical (Northridge, CA, USA) in which a mixture of a patient’s own cells is sprayed directly to the site for wounds, scars and hypopigmentation [47]. This system provides a rapid way to collect the patient’s cells and deliver them to a much larger surface area without culturing the cells. It resulted in accelerated healing comparable to skin grafting with decreased wound dressing time, and less scarring.

## 4. Therapeutic Indications

### 4.1. Cutaneous Burns

In cutaneous burns the goals are to restore barrier function, prevent infection, minimize scarring, and prevent disfigurement. Wound dressings and synthetic substitutes provide a temporary barrier to minimize infection [48]. However, wound care cannot fully replace the normal skin barrier, and burns often result in scarring leading to psychological distress [49]. Skin transplants from split- or full-thickness grafts are well-established techniques for restoring lost skin [50,51]. However, donor skin availability and the size of the area involved have led to the development of techniques to expand cells *in vitro* for generation of engineered full thickness tissue substitutes. Caruso and colleagues understood the limitation of epithelial autografts in treating burn wounds and developed a fibroblast-keratinocyte composite from a burn victim’s normal skin. This composite could be used to cover third degree burns [52]. In another report, a 19 years old patient suffering from a third degree burn involving 76% of the body surface area was treated with a cultured autologous fibroblast and keratinocytes suspension after stabilizing the burn area with a commercial dermis equivalent. The patient exhibited improved wound closure with the development of functional skin over a one year follow up [53]. The bi-layered skin substitute developed by Boyce and colleagues from autologous fibroblasts and keratinocytes resulted in faster wound closure and decreased the requirements for donor skin harvesting for additional grafting in full-thickness burns [45]. Another report of autologous bioengineered skin used in two patients with extensive burns resulted in the restoration of bi-layered skin that was maintained after two years of follow up [54]. The feasibility of using artificial skin from autologous fibroblasts and keratinocytes for an indication other than burns was demonstrated by Llames *et al*. They treated patients with large wounds from the removal of giant nevi with bio-engineered skin. In these patients, the bio-engineered skin resulted in permanent re-epithelialization in all cases without blistering or skin retractions [55]. These studies show that autologous fibroblast therapies alone or as part of bio-engineered skin equivalents are effective in burns and other large epithelial defects.

### 4.2. Cosmetic Indications

Facial contour deformities like nasolabial fold wrinkles and acne scars may impair skin function and can cause psychological discomfort leading to decreased quality of life [56,57]. Dermal fillers are one of the main therapeutic approaches for soft tissue augmentation, but complications such as bruising, unwanted swelling, skin dyspigmentation, skin infections, and subcutaneous nodules can occur [58]. Not surprisingly, an alternative using autologous fibroblasts as natural filler is available to generate matrix proteins such as collagen. West and Alster described the concept that injecting autologous dermal fibroblasts could replace intradermal injection of silicone and other fillers for treating facial wrinkles. This novel approach has the potential to avoid hypersensitivity reactions associated with dermal fillers and result in a sustained therapeutic effect [1,59,60]. In a pilot study, Watson and colleagues showed that nine out of ten patients who exhibited facial rhytides showed some improvement when autologous fibroblasts were injected in the target site, and increases in collagen were noted in the dermis [61]. In studies using autologous fibroblast therapy in which 20 million/mL autologous cells were injected into the dermis in three doses spaced one month apart, significant improvement in facial contour was observed. In addition to improvement of facial rhytides, acne scars also improved with no adverse events when studied [62]. Given the early successes, multicenter placebo control trials for the treatment of nasolabial fold wrinkles and facial contour deformities with autologous fibroblast were expanded to develop an aesthetic indication [43]. These studies led to the approval in 2011 of LAVIV™, an autologous cellular therapy product manufactured by Fibrocell Science, Inc. (Exton, PA, USA) for use in the treatment of nasolabial wrinkles. The use of autologous fibroblasts has since expanded to acne scars [63] and peri-orbital skin flaccidity [64].

### 4.3. Orthopedic Indications

Replicel is developing autologous fibroblast therapy from fibroblasts isolated from the dermal sheath (DS) surrounding hair follicles to treat chronic tendinosis. A randomized, double-blind study was performed in thirty-two patients with Achilles tendon injuries who were treated with autologous skin-derived fibroblasts [65]. Connell and colleagues has previously established the safety and efficacy of fibroblast injection in refractory lateral epicondylitis [66]. The fibroblast therapy for tendinosis was found to be safe and showed significant improvement in healing the injured site in short term and long term studies. A related orthopedic indication is the use of autologous fibroblasts in osseointegrative prostheses. Autologous fibroblasts have been shown to reduce skin infection and osteomyelitis, one of the most troubling complications at the prosthesis attachment site, when applied to the implant surface [67].

### 4.4. Wound Repair

Regenerating skin after surgical wounds is another application for autologous fibroblasts. Wounds treated with autologous dermal grafts in hyaluronic acid sheets re-epithelialized faster and patient satisfaction and scar appearance were improved compared to skin graft alone. These findings were studied in skin defects after the removal of basal cell carcinomas [68].

Apligraft (Organogenesis, Canton, OH, USA) is an allograft consisting of bi-layered skin derived from cultured fibroblasts and keratinocytes isolated from foreskin. It is FDA approved for chronic wound conditions like venous leg ulcers and diabetic foot ulcers [69]. However it is an allogeneic product and is prone to immune-mediated rejection. This product could be improved by the use of autologous skin constituents consisting of fibroblasts and keratinocytes to generate a “custom” bi-layered skin substitutes for skin repair and regeneration. Along these lines, proof of principle experiments have shown that long standing diabetic ulcers treated with a mixture of autologous fibroblast and keratinocytes in a fibrin suspension resulted in faster healing of ulcers with no adverse effect [44].

## 5. Regional and Microanatomic Specificity of Fibroblast Populations

### 5.1. Regional Identity

One of the most intriguing properties of skin is its regional specificity. Regional specificity is observed in species from birds to humans and reflects an adaptive trait that is rigidly maintained. In humans, this phenomenon is readily appreciated in the differences between hair-bearing and non-hair bearing skin. However, many other examples exist such as differences between palmar-plantar (volar) skin of the palm and sole of the hands and feet respectively and non-volar skin of the face. An interesting corollary in birds is the presence of feathers *vs*. scales. This regional skin variation provides an opportunity for understanding the developmental mechanisms that determine tissue phenotype and raises a possible platform for therapeutic intervention for regional skin diseases.

One of the most studied areas of regional skin variation is hair-bearing skin. Androgenetic alopecia is estimated to affect 50% of the male and female population by 50 years of age [70]. Therapies such as minoxidil, finasteride, and spironolactone have been shown to slow the progression of androgenetic alopecia with mixed results. Hormonal sensitivity is also implicated in excessive facial hair (hirsutism) in women, while other investigators have implicated the disruption in pathways such as Prostaglandin D2 in hair loss [71]. However, despite continued research into hair loss, one of the most effective treatments remains hair transplantation.

In animals, classic transplantation experiments swapped the epidermis and dermis of different parts of birds to see which controlled the other. Normally a bird has scales on its feet and feathers on its wing. In birds, the scales and feathers are made by the epidermal keratinocytes. Early investigators exchanged the dermis and epidermis from the foot and wing to determine in mismatched tissue whether the epidermis maintained its original product (scales or feathers) or whether the dermis was responsible for “instructing” the epidermis’ unique tissue identity. Those studies demonstrated that the dermis controls the epidermal phenotype. In these experiments, mismatched epidermis would switch from feather to scale, if foot dermis were added to wing epidermis [72]. This series of experiments demonstrated that fibroblasts could “induce” phenotype, *i.e*., feathers *vs*. scale. The researchers were able to induce feathers on the foot and scales on the wing of manipulated birds.

Human studies have demonstrated similar results; scalp dermal fibroblasts create long hairs if transplanted to the arm [73] because fibroblasts can “remember” their position in the body and stimulate the corresponding phenotype. When human dermal fibroblasts are removed from different areas of the skin and their gene expression is measured, investigators have found persistent expression of specific genes that reflect the location of origin of the fibroblasts [40,74,75]. The most prominent site-specific genes are the *HOX* or *homeobox* genes. Fibroblasts retained their *HOX* signatures even after 35 cell doublings in cell culture [76].

Preliminary studies using human cells have been published which demonstrate the importance of volar fibroblasts in controlling volar (palmar-plantar) gene expression [77]. For example, Yamaguchi and colleagues demonstrated that human non-volar (thigh) keratinocytes when transplanted onto human palms began to express *KRT9*. Although initially devoid of *KRT9* expression, by three weeks post-transplantation onto volar dermis, the non-volar trunk keratinocytes express some *KRT9*. By five weeks post-translation, all transplanted keratinocytes expressed *KRT9*. Coincident with *KRT9* expression was the adoption of other volar skin features such as a thick stratum corneum.

Rinn and colleagues [75] extended these findings by replicating results showing that *in vitro* human volar fibroblasts can induce *KRT9* expression in cultured non-volar keratinocytes. Through an analysis of microarray data on cultured fibroblasts from volar locations, they were able to demonstrate that the *KRT9* induction required the homeobox gene *HoxA13* and its downstream mediator *Wnt5a*. In fact, they were able to show that *Wnt5a* itself was sufficient to induce *KRT9* gene expression. This demonstrated that canonical developmental genes are responsible for a given phenotype. Another series of studies by Yamaguchi and colleagues [78,79] describes the ability of *DKK1* to increase epidermal thickness and the expression of *KRT9* and induce melanocytes. These studies corroborate the findings in birds that dermal fibroblasts control tissue identity of the epidermis. They further demonstrate that fibroblasts retain their *HOX* expression despite expansion in culture thereby supporting the idea that fibroblast retain their functional identity after multiplication in culture.

In summary, dermal fibroblasts do not merely play a supportive role in the skin. Instead, fibroblasts exert a major influence in determining the regional identity of skin. The importance of fibroblasts in developmental biology as inductive cells critical to defining regional phenotypes raises the potential for the use of fibroblasts to manipulate skin characteristics in cellular therapy applications.

#### 5.1.1. Induction of Acral Skin

In current clinical practice, autologous fibroblast transplants are commonplace in the form of split-thickness-skin grafts or partial-thickness-skin grafts. These grafts contain both epidermis and a component of the fibroblast-rich dermis. Allogeneic fibroblast treatments occur routinely in wound care centers in the form of cultured cells from human foreskin. This therapy (Apligraf) is FDA approved for the treatment of diabetic and venous foot ulcers [80–83]. The newest application of fibroblast therapy was the approval by the FDA in June 2011 of cultured autologous fibroblasts as skin filler for unwanted facial skin wrinkles. This product is marketed as LAVIV™ (Fibrocell Sciences) [84]. Cultured autologous fibroblast therapy appears safe and effective based on the FDA approval of LAVIV™. Fibrocell Sciences has development underway of cultured autologous fibroblasts for acne scars, restrictive burn scars, vocal cord scarring, and tendon repair. Replicel has expanded its autologous product research and development into hair regeneration, skin aging, and tendinosis, but does not yet have FDA approval for a cultured autologous fibroblast product.

The use of autologous cultured fibroblasts for amputees was initially proposed by one of the authors (Dr. Meyerle) as a way to help amputees with skin breakdown and disease at the stump site. Given that skin disease affects up to 48% of amputees at the stump site [85], the goal of cultured autologous fibroblast cell therapy is to alter the phenotype of the stump skin to better support the prosthetic device. In essence, cultured autologous fibroblasts could be employed to “volarize” the stump skin such that the stump takes on the characteristics of the skin found on the palms and soles. While cultured autologous fibroblast therapies are promising, the logistics of collection and transportation and the production costs are limiting. However, as these technical hurdles are overcome, autologous fibroblasts have increasing promise as tools for regenerative medicine.

**Figure 2.**
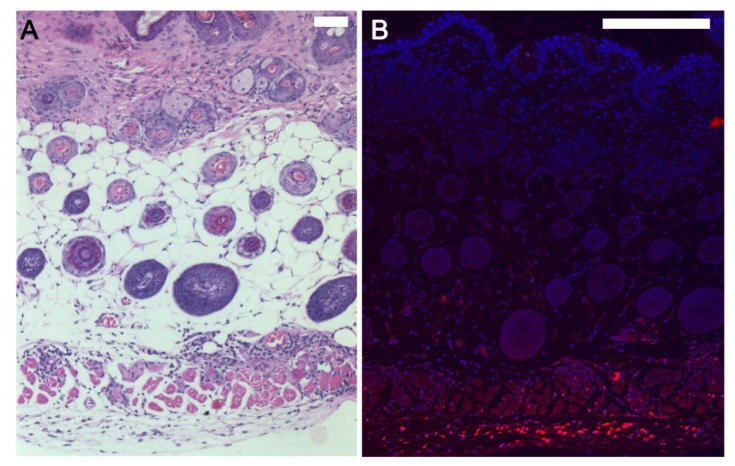
Human fibroblasts injected into the hypodermis of mice survive for at least 8 weeks. (**A**) H&E stained sections shows the presence of injected cells visible as a non-native cluster of fibroblasts in the hypodermis below the muscle layer; (**B**) Fibroblasts were incubated with red dye tracer, CellTracker™Red CMTPX and are bright red in serial section evaluated using a fluorescence microscope confirming the identity, implantation and survival of human cells. Nuclei stained with DAPI. Scale bars: **A**, 130 μm and **B**, 250 μm.

As a proof of principle we evaluated the longevity, persistence and ease of locating the injected human fibroblasts in skin using a mouse xenograft model. We isolated fibroblasts from foreskins of unidentified normal neonates. Briefly, tissues were treated overnight with dispase at 4 °C and epidermal sheets were separated from dermis and the dermal layer was cut into small pieces and placed in DMEM with 10% FBS in culture dishes. Media was changed twice weekly until the fibroblasts migrated out to cover the dishes. Cells were then harvested for passage. Some fibroblast cultures were incubated with red dye tracer, CellTracker™Red CMTPX before the experiment. Six to eight weeks old Cr:NIH(S)-nu/nu female mice were injected hypodermally with one million fibroblasts in 100 μL of 1:1 DMEM/F12 media. The longevity of these fibroblasts was evaluated by harvesting and analyzing the injection site after 1, 2, 4 or 8 weeks. Human fibroblasts were present for at least 8 weeks after injection (Figure 2), with or without tracers. Human fibroblasts were tightly clustered at the injection site at 1 week, and it appeared that cells migrated away from each other and became increasingly dispersed at 4 and 8 weeks. We confirmed the presence of transplanted cells by: (1) histologically the injected cells are visible in H&E stained sections as a non-native cluster of fibroblasts in the hypodermis; and (2) fluorescence microscopy method showed the cells labeled with a tracer are bright red in sections at the injection site (Figure 2). These results suggest that injected fibroblasts have the potential to survive for durations sufficient to modify their local microenvironment.

### 5.2. Microanatomic Specificity

#### 5.2.1. Papillary and Reticular Dermal Fibroblasts

**Figure 3.**
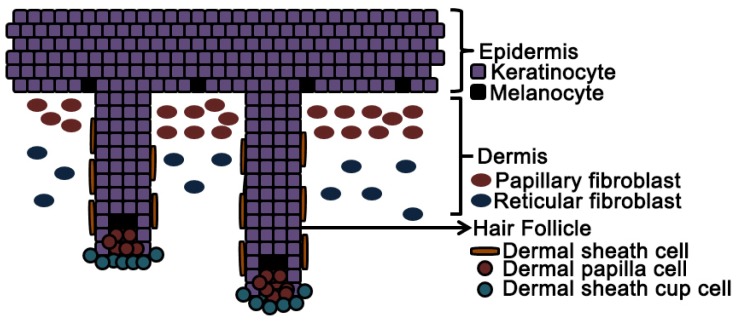
Microanatomic regions of the skin have distinct fibroblast populations which differ in their physical characteristics and functional capabilities.

In addition to the regional identify of fibroblasts isolated from different anatomical locations, it is now appreciated that fibroblasts from different microanatomic regions have properties specific to their particular niche [29]. Based on the sub anatomic location in the skin, fibroblasts can be divided into hair follicle associated fibroblasts, including dermal sheath and dermal papilla cells and interstitial fibroblasts, comprising papillary and reticular dermal fibroblasts (Figure 3).

Harper and Grove, realizing the functional and structural differences between reticular and papillary dermis, showed that the fibroblast like cells isolated from these regions proliferate at a differential rate [86]. During normal aging, the papillary dermis skin is shown to atrophy as the reticular fibroblasts begin to occupy the papillary dermis. This is thought to impact epithelial morphogenesis and regeneration [87]. Fibroblasts isolated from papillary or reticular dermis have different genetic markers and functional properties demonstrating the regional uniqueness of fibroblast sub-populations [88–90]. Collagen synthesis was found to be similar regardless of fibroblast origin in the dermis [91], however consistently increased amounts of pro-collagen was secreted by reticular fibroblasts and not by papillary fibroblasts. Functionally, papillary fibroblasts have enhanced epidermal differentiation and maturation and secrete different soluble factors in reconstituted skin models [90] when compared with reticular fibroblasts. Conversely, reticular fibroblasts secrete different levels cytokines such as keratinocyte growth factor and interleukin-6, than papillary fibroblasts, and suppresses terminal differentiation of keratinocytes as well the formation of the basement membrane zone when incorporated into skin equivalents [92]. Reticular fibroblasts also secrete molecular targets that differentially remodel the collagen-glycosaminoglycan matrix [93]. These findings strongly suggest that the papillary and reticular dermis have two distinct fibroblast populations. Recently, Driskell and colleagues discovered two distinct lineages of fibroblast in skin and elegantly demonstrated that the upper dermal lineage is essential for hair follicle formation. Lower dermal lineage which is mainly responsible for synthesizing extra cellular matrix initiates the wound healing process, thereby resulting in matrix rich tissue devoid of hair follicles [29]. These studies may have potential implications for the field of autologous fibroblast therapies for anti-aging in addition to more common applications to wound healing.

#### 5.2.2. Dermal Papilla and Dermal Sheath Cells

Hair follicle associated mesenchymal cells namely dermal sheath cells (DS, located in areas surrounding the outside of hair follicle) and dermal papilla (DP, located at the root of the hair follicle) are progenitor cells [94,95] that can differentiate into dermal fibroblast during wound healing, induce hair follicle neogeneis, and regenerate dermal layer of the skin (Figure 3). Blood vessels in the skin are also associated with fibroblasts namely perivascular cells [96], however, these are not discussed in this review.

The identification and culture of DP cells, distinct fibroblast cell populations found at the base of the hair follicle and the subsequent demonstration of their hair inducing capabilities [73,97–99] opened a new field of cellular therapy for hair follicle neogenesis, and for treating alopecia and other hair loss disorders [100,101]. DP and DS cells are functionally different from dermal fibroblasts as they exhibit variance in the localization of immune cells [102] and differential activation of NFκB and decreased sensitivity towards bacterial lipopolysaccharide [102,103]. A landmark observation in human hair follicle neogenesis was made when Reynolds and colleagues transplanted male DS cells from the scalp into the forearm of a female human subject. This experiment demonstrated that hair growth was partially dependent on DS cells for formation of a DP [104]. Recently, fibroblast cells isolated from the peri-bulbar DS have also been shown to induce hair follicles [105]. These studies demonstrate that only dermal fibroblasts from specific locations in the skin have the capacity to induce hair neogenesis.

#### 5.2.3. Induction of Hair Follicles

**Figure 4.**
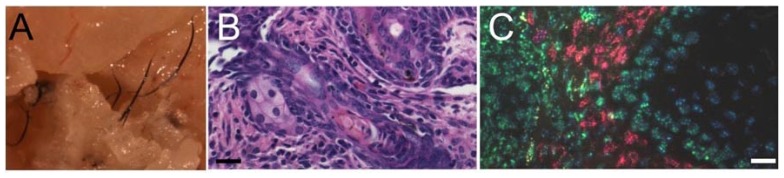
Human dermal papilla (DP) spheroids induce chimeric hair follicles in reconstitution assay. (**A**) Hair fibers in the hypodermis 4 weeks following injection of a mixture of human DP spheroids (10,000 cells/spheroid) and mouse epidermal cells; (**B**) H&E stained horizontal section of injection cyst forming HFs and sebaceous glands; and (**C**) Fluorescence in situ hybridization analysis of the assay site reveals that human DP cells probed with human specific pan-centromeric probe (red) co-exist with the mouse epidermal cells probed with mouse specific pan-centromeric probe (green). Scale bars: **A**, **B** and **C**, 35 μm.

Isolated DP cells including peri-bulbar sheath cells and DS cells are being studied extensively for their hair inducing properties which could be translated into cellular therapy for regenerating hair follicles. Cells with the potential for hair follicle neogenesis can be either injected directly in the bald scalp or in areas where hair follicles are desired or can be incorporated in skin substitutes to regenerate skin appendages. One limitation of applying these cells for hair regeneration is that expansion and frequent passage of trichogenic cells results in loss of hair inductive potential. Advancements in cell culture technique like using less trypsin during multiplication [106], three dimensional culture systems [107,108], xeno free culture system [109], conditioning with human serum [63,110] or supplementing media with morphogens like wnt and bmp modulators [111–113] can lead to methods for extending the efficacy of these cells. We used the reconstitution assay [114,115] with minor modifications to evaluate whether the trichogenic potential of DP cells can be maintained by using three dimensional culture systems. Unlike neonatal mouse dermal cells, human DP cells grown in monolayer culture failed to induce HFs when injected as a single cell suspension mixed with mouse epidermal aggregates. In contrast, human DP cells grown as spheroids induced HFs when coinjected with mouse epidermal aggregates. The resultant HFs contained hair shafts and sebaceous glands (Figure 4A,B), similar to the results of others [115]. As expected, HFs were chimeric with mouse-derived cells comprising the epithelial component and human-derived cells dominating the region adjacent to the epithelium (Figure 4C).

We have also recently demonstrated for the first time that isolated, cultured and dissociated human DP cells can induce complete human hair follicles when combined with neonatal foreskin keratinocytes and grafted onto a nude mice [116]. The clinical development of hair follicle inducing dermal cells as a cellular therapy for treating alopecia is being pursued by Replicel Life Sciences Incorporated (Vancouver, BC, Canada) [117]. Replicel, is conducting a clinical trial (clinicaltrials.gov identifier: NCT01286649) using the autologous cultured dermal sheath cup cells isolated from a biopsy from the occipital scalp for the treatment of androgenetic alopecia in humans. Dermal sheath cup cells are located in the peribulbar region of hair follicles (Figure 3) and have lower alkaline phosphatase activity than DP cells. Dermal sheath cup cells appear to be similar to DP cells in their hair inducing capacity [105]. In an ongoing phase 1/2a clinical safety trial, majority of the injections of dermal sheath cup cells were well tolerated after a six months follow up for safety and no serious adverse events were reported 12 months after injections. After 6 months more patients demonstrated an increase in hair growth efficacy and Replicel is on track for a phase 2 dosing trial. Aderans Research Institute Incorporated (ARI, Marietta, GA, USA) Aderans Research Institute has completed a clinical trial (clinicaltrials.gov identifier: NCT01669746) for male and female pattern baldness using autologous cell isolated from patient’s skin.

## 6. Conclusions

Cellular therapy based on autologous dermal fibroblast holds enormous promise to the field of regeneration medicine. It offers a safe, immunologically acceptable and simple alternative for tissue regeneration applications. Basic research into the mechanism(s) of regional phenotype determination by fibroblasts will help to develop reprogramming options for cellular therapy applications. Practical challenges including scale up, formulation, storage, transport and delivery mechanisms are the main challenges to development and clinical translation of preclinical observations. However, recent FDA approval of fibroblast therapy applications for nasolabial folds and the expanding pipeline of other fibroblast therapies in clinical development show that cellular therapy is an expanding field.

## Abbreviations

DP: dermal papilla
DS: dermal sheath
FGF: fibroblast growth factor 2
GMP: Good Manufacturing Process
HGF: hepatocyte growth factor
ILF: integrin-linked kinase
MMP: Matrix metalloproteinase
VEGF: vascular endothelial growth factor

## References

[1] Weiss R.A. (2013). Autologous cell therapy: Will it replace dermal fillers?. Fac. Plast. Surg. Clin. N. Am.

[2] McHeik J.N., Barrault C., Pedretti N., Garnier J., Juchaux F., Levard G., Morel F., Lecron J.C., Bernard F.X. (2013). Foreskin-isolated keratinocytes provide successful extemporaneous autologous paediatric skin grafts. J. Tissue Eng. Regener. Med.

[3] Asai J., Takenaka H., Ii M., Asahi M., Kishimoto S., Katoh N., Losordo D.W. (2013). Topical application of *ex vivo* expanded endothelial progenitor cells promotes vascularisation and wound healing in diabetic mice. Int. Wound J.

[4] Wu Y., Zhao R.C., Tredget E.E. (2010). Concise review: Bone marrow-derived stem/progenitor cells in cutaneous repair and regeneration. Stem Cells.

[5] Ohyama M., Okano H. (2014). Promise of human induced pluripotent stem cells in skin regeneration and investigation. J. Investig. Dermatol.

[6] Lohmeyer J.A., Liu F., Kruger S., Lindenmaier W., Siemers F., Machens H.G. (2011). Use of gene-modified keratinocytes and fibroblasts to enhance regeneration in a full skin defect. Langenbeck’s Arch. Surg. Dtsch. Ges. Chir.

[7] Hata K. (2007). Current issues regarding skin substitutes using living cells as industrial materials. J. Artif. Organs.

[8] Greaves N.S., Iqbal S.A., Baguneid M., Bayat A. (2013). The role of skin substitutes in the management of chronic cutaneous wounds. Wound Repair Regen.

[9] Catalano E., Cochis A., Varoni E., Rimondini L., Azzimonti B. (2013). Tissue-engineered skin substitutes: An overview. J. Artif. Organs.

[10] Greaves N.S., Ashcroft K.J., Baguneid M., Bayat A. (2013). Current understanding of molecular and cellular mechanisms in fibroplasia and angiogenesis during acute wound healing. J. Dermatol. Sci.

[11] Vedrenne N., Coulomb B., Danigo A., Bonte F., Desmouliere A. (2012). The complex dialogue between (myo)fibroblasts and the extracellular matrix during skin repair processes and ageing. Pathologie-Biologie.

[12] Glim J.E., Niessen F.B., Everts V., van Egmond M., Beelen R.H. (2013). Platelet derived growth factor-CC secreted by M2 macrophages induces alpha-smooth muscle actin expression by dermal and gingival fibroblasts. Immunobiology.

[13] McGee H.M., Schmidt B.A., Booth C.J., Yancopoulos G.D., Valenzuela D.M., Murphy A.J., Stevens S., Flavell R.A., Horsley V. (2013). IL-22 promotes fibroblast-mediated wound repair in the skin. J. Investig. Dermatol.

[14] Shi H.X., Lin C., Lin B.B., Wang Z.G., Zhang H.Y., Wu F.Z., Cheng Y., Xiang L.J., Guo D.J., Luo X. (2013). The anti-scar effects of basic fibroblast growth factor on the wound repair *in vitro* and *in vivo*. PLoS One.

[15] Ko J., Jun H., Chung H., Yoon C., Kim T., Kwon M., Lee S., Jung S., Kim M., Park J.H. (2011). Comparison of EGF with VEGF non-viral gene therapy for cutaneous wound healing of streptozotocin diabetic mice. Diabetes Metab. J.

[16] Loyd C.M., Diaconu D., Fu W., Adams G.N., Brandt E., Knutsen D.A., Wolfram J.A., McCormick T.S., Ward N.L. (2012). Transgenic overexpression of keratinocyte-specific VEGF and Ang1 in combination promotes wound healing under nondiabetic but not diabetic conditions. Int. J. Clin. Exp. Pathol.

[17] Kellouche S., Mourah S., Bonnefoy A., Schoevaert D., Podgorniak M.P., Calvo F., Hoylaerts M.F., Legrand C., Dosquet C. (2007). Platelets, thrombospondin-1 and human dermal fibroblasts cooperate for stimulation of endothelial cell tubulogenesis through VEGF and PAI-1 regulation. Exp. Cell Res.

[18] Conway K., Price P., Harding K.G., Jiang W.G. (2006). The molecular and clinical impact of hepatocyte growth factor, its receptor, activators, and inhibitors in wound healing. Wound Repair Regen.

[19] Schnickmann S., Camacho-Trullio D., Bissinger M., Eils R., Angel P., Schirmacher P., Szabowski A., Breuhahn K. (2009). AP-1-controlled hepatocyte growth factor activation promotes keratinocyte migration via CEACAM1 and urokinase plasminogen activator/urokinase plasminogen receptor. J. Investig. Dermatol.

[20] Serrano I., Diez-Marques M.L., Rodriguez-Puyol M., Herrero-Fresneda I., Raimundo Garcia del M., Dedhar S., Ruiz-Torres M.P., Rodriguez-Puyol D. (2012). Integrin-linked kinase (ILK) modulates wound healing through regulation of hepatocyte growth factor (HGF). Exp. Cell Res.

[21] Finnson K.W., McLean S., di Guglielmo G.M., Philip A. (2013). Dynamics of transforming growth factor beta signaling in wound healing and scarring. Adv. Wound Care.

[22] Rolfe K.J., Irvine L.M., Grobbelaar A.O., Linge C. (2007). Differential gene expression in response to transforming growth factor-β1 by fetal and postnatal dermal fibroblasts. Wound Repair Regen.

[23] Gill S.E., Parks W.C. (2008). Metalloproteinases and their inhibitors: Regulators of wound healing. Int. J. Biochem. Cell Biol.

[24] Olaso E., Lin H.C., Wang L.H., Friedman S.L. (2011). Impaired dermal wound healing in discoidin domain receptor 2-deficient mice associated with defective extracellular matrix remodeling. Fibrogen. Tissue Repair.

[25] Gawronska-Kozak B. (2011). Scarless skin wound healing in FOXN1 deficient (nude) mice is associated with distinctive matrix metalloproteinase expression. Matrix Biol. J. Int. Soc. Matrix Biol.

[26] Bouissou H., Pieraggi M., Julian M., Uhart D., Kokolo J. (1988). Fibroblasts in dermal tissue repair. Electron microscopic and immunohistochemical study. Int. J. Dermatol.

[27] Purdue G.F., Hunt J.L., Still J.M., Law E.J., Herndon D.N., Goldfarb I.W., Schiller W.R., Hansbrough J.F., Hickerson W.L., Himel H.N. (1997). A multicenter clinical trial of a biosynthetic skin replacement, Dermagraft-TC, compared with cryopreserved human cadaver skin for temporary coverage of excised burn wounds. J. Burn Care Rehabil.

[28] Junker J.P., Sommar P., Skog M., Johnson H., Kratz G. (2010). Adipogenic, chondrogenic and osteogenic differentiation of clonally derived human dermal fibroblasts. Cells Tissues Organs.

[29] Driskell R.R., Lichtenberger B.M., Hoste E., Kretzschmar K., Simons B.D., Charalambous M., Ferron S.R., Herault Y., Pavlovic G., Ferguson-Smith A.C. (2013). Distinct fibroblast lineages determine dermal architecture in skin development and repair. Nature.

[30] Keller G., Sebastian J., Lacombe U., Toft K., Lask G., Revazova E. (2000). Safety of injectable autologous human fibroblasts. Bull. Exp. Biol. Med.

[31] Lamme E.N., van Leeuwen R.T., Brandsma K., van Marle J., Middelkoop E. (2000). Higher numbers of autologous fibroblasts in an artificial dermal substitute improve tissue regeneration and modulate scar tissue formation. J. Pathol.

[32] Svensjo T., Yao F., Pomahac B., Winkler T., Eriksson E. (2002). Cultured autologous fibroblasts augment epidermal repair. Transplantation.

[33] Morimoto N., Saso Y., Tomihata K., Taira T., Takahashi Y., Ohta M., Suzuki S. (2005). Viability and function of autologous and allogeneic fibroblasts seeded in dermal substitutes after implantation. J. Surg. Res.

[34] Lamme E.N., van Leeuwen R.T., Mekkes J.R., Middelkoop E. (2002). Allogeneic fibroblasts in dermal substitutes induce inflammation and scar formation. Wound Repair Regen.

[35] Hong L., Mao J.J. (2004). Tissue-engineered rabbit cranial suture from autologous fibroblasts and BMP2. J. Dent. Res.

[36] Liu W., Chen B., Deng D., Xu F., Cui L., Cao Y. (2006). Repair of tendon defect with dermal fibroblast engineered tendon in a porcine model. Tissue Eng.

[37] Kanzaki M., Yamato M., Yang J., Sekine H., Takagi R., Isaka T., Okano T., Onuki T. (2008). Functional closure of visceral pleural defects by autologous tissue engineered cell sheets. Eur. J. Cardio-Thorac. Surg.

[38] Egan J.J., Saltis J., Wek S.A., Simpson I.A., Londos C. (1990). Insulin, oxytocin, and vasopressin stimulate protein kinase C activity in adipocyte plasma membranes. Proc. Natl. Acad. Sci. USA.

[39] Van Zuijlen P.P., van Leeuwen R.T., Middelkoop E. (1998). Practical sources for autologous fibroblasts to prepare a bioengineered dermal equivalent. Burns.

[40] Rinn J.L., Bondre C., Gladstone H.B., Brown P.O., Chang H.Y. (2006). Anatomic demarcation by positional variation in fibroblast gene expression programs. PLoS Genet.

[41] Johansson J.A., Headon D.J. (2014). Regionalisation of the skin. Sem. Cell Dev. Biol.

[42] Liu J.Y., Hafner J., Dragieva G., Burg G. (2006). High yields of autologous living dermal equivalents using porcine gelatin microbeads as microcarriers for autologous fibroblasts. Cell Transpl.

[43] Smith S.R., Munavalli G., Weiss R., Maslowski J.M., Hennegan K.P., Novak J.M. (2012). A multicenter, double-blind, placebo-controlled trial of autologous fibroblast therapy for the treatment of nasolabial fold wrinkles. Dermatol. Surg.

[44] Marcelo D., Beatriz P.M., Jussara R., Fabiana B. (2012). Tissue therapy with autologous dermal and epidermal culture cells for diabetic foot ulcers. Cell Tissue Bank.

[45] Boyce S.T., Kagan R.J., Greenhalgh D.G., Warner P., Yakuboff K.P., Palmieri T., Warden G.D. (2006). Cultured skin substitutes reduce requirements for harvesting of skin autograft for closure of excised, full-thickness burns. J. Trauma.

[46] Scuderi N., Onesti M.G., Bistoni G., Ceccarelli S., Rotolo S., Angeloni A., Marchese C. (2008). The clinical application of autologous bioengineered skin based on a hyaluronic acid scaffold. Biomaterials.

[47] Gilleard O., Segaren N., Healy C. (2013). Experience of recell in skin cancer reconstruction. Arch. Plast. Surg.

[48] Vogt P.M., Kolokythas P., Niederbichler A., Knobloch K., Reimers K., Choi C.Y. (2007). Innovative wound therapy and skin substitutes for burns. Chirurg.

[49] Blais M., Parenteau-Bareil R., Cadau S., Berthod F. (2013). Concise review: Tissue-engineered skin and nerve regeneration in burn treatment. Stem Cells Transl. Med.

[50] Alexander J.W., MacMillan B.G., Law E., Kittur D.S. (1981). Treatment of severe burns with widely meshed skin autograft and meshed skin allograft overlay. J. Trauma.

[51] Killat J., Reimers K., Choi C.Y., Jahn S., Vogt P.M., Radtke C. (2013). Cultivation of keratinocytes and fibroblasts in a three-dimensional bovine collagen-elastin matrix (Matriderm(R)) and application for full thickness wound coverage *in vivo*. Int. J. Mol. Sci..

[52] Caruso D.M., Schuh W.H., Al-Kasspooles M.F., Chen M.C., Schiller W.R. (1999). Cultured composite autografts as coverage for an extensive body surface area burn: Case report and review of the technology. Burns.

[53] Wisser D., Steffes J. (2003). Skin replacement with a collagen based dermal substitute, autologous keratinocytes and fibroblasts in burn trauma. Burns.

[54] Llames S.G., del Rio M., Larcher F., Garcia E., Garcia M., Escamez M.J., Jorcano J.L., Holguin P., Meana A. (2004). Human plasma as a dermal scaffold for the generation of a completely autologous bioengineered skin. Transplantation.

[55] Llames S., Garcia E., Garcia V., del Rio M., Larcher F., Jorcano J.L., Lopez E., Holguin P., Miralles F., Otero J. (2006). Clinical results of an autologous engineered skin. Cell Tissue Bank.

[56] Lee I.S., Lee A.R., Lee H., Park H.J., Chung S.Y., Wallraven C., Bulthoff I., Chae Y. (2014). Psychological distress and attentional bias toward acne lesions in patients with acne. Psychol. Health Med.

[57] Griffiths C., Williamson H., Rumsey N. (2012). The romantic experiences of adolescents with a visible difference: Exploring concerns, protective factors and support needs. J. Health Psychol.

[58] Funt D., Pavicic T. (2013). Dermal fillers in aesthetics: An overview of adverse events and treatment approaches. Clin. Cosmet. Investig. Dermatol.

[59] West T.B., Alster T.S. (1998). Autologous human collagen and dermal fibroblasts for soft tissue augmentation. Dermatol. Surg.

[60] Boss W.K., Usal H., Fodor P.B., Chernoff G. (2000). Autologous cultured fibroblasts: A protein repair system. Ann. Plast. Surg.

[61] Watson D., Keller G.S., Lacombe V., Fodor P.B., Rawnsley J., Lask G.P. (1999). Autologous fibroblasts for treatment of facial rhytids and dermal depressions. A pilot study. Arch. Fac. Plast. Surg.

[62] Weiss R.A., Weiss M.A., Beasley K.L., Munavalli G. (2007). Autologous cultured fibroblast injection for facial contour deformities: A prospective, placebo-controlled, Phase III clinical trial. Dermatol. Surg.

[63] Munavalli G.S., Smith S., Maslowski J.M., Weiss R.A. (2013). Successful treatment of depressed, distensible acne scars using autologous fibroblasts: A multi-site, prospective, double blind, placebo-controlled clinical trial. Dermatol. Surg.

[64] Eca L.P., Pinto D.G., de Pinho A.M., Mazzetti M.P., Odo M.E. (2012). Autologous fibroblast culture in the repair of aging skin. Dermatol. Surg.

[65] Obaid H., Clarke A., Rosenfeld P., Leach C., Connell D. (2012). Skin-derived fibroblasts for the treatment of refractory Achilles tendinosis: Preliminary short-term results. J. Bone Jt. Surg. Am. Vol.

[66] Connell D., Datir A., Alyas F., Curtis M. (2009). Treatment of lateral epicondylitis using skin-derived tenocyte-like cells. Br. J. Sports Med.

[67] Shevtsov M.A., Galibin O.V., Yudintceva N.M., Blinova M.I., Pinaev G.P., Ivanova A.A., Savchenko O.N., Suslov D.N., Potokin I.L., Pitkin E. (2013). Two-stage implantation of the skin- and bone-integrated pylon seeded with autologous fibroblasts induced into osteoblast differentiation for direct skeletal attachment of limb prostheses. J. Biomed. Mater. Res. A.

[68] Han S.K., Kim S.Y., Choi R.J., Jeong S.H., Kim W.K. (2014). Comparison of tissue-engineered and artificial dermis grafts after removal of basal cell carcinoma on face-A pilot study. Dermatol. Surg.

[69] Karr J.C. (2011). Retrospective comparison of diabetic foot ulcer and venous stasis ulcer healing outcome between a dermal repair scaffold (PriMatrix) and a bilayered living cell therapy (Apligraf). Adv. Skin Wound Care.

[70] Rogers N.E., Avram M.R. (2008). Medical treatments for male and female pattern hair loss. J. Am. Acad. Dermatol.

[71] Nieves A., Garza L.A. (2014). Does Prostaglandin D_2_ hold the cure to male pattern baldness?. Exp. Dermatol.

[72] Dhouailly D. (1973). Dermo-epidermal interactions between birds and mammals: Differentiation of cutaneous appendages. J. Embryol. Exp. Morphol.

[73] Jahoda C.A., Horne K.A., Oliver R.F. (1984). Induction of hair growth by implantation of cultured dermal papilla cells. Nature.

[74] Chang H.Y., Chi J.T., Dudoit S., Bondre C., van de Rijn M., Botstein D., Brown, P.O. (2002). Diversity, topographic differentiation, and positional memory in human fibroblasts. Proc. Natl. Acad. Sci. USA.

[75] Rinn J.L., Wang J.K., Allen N., Brugmann S.A., Mikels A.J., Liu H., Ridky T.W., Stadler H.S., Nusse R., Helms J.A. (2008). A dermal HOX transcriptional program regulates site-specific epidermal fate. Genes Dev.

[76] Wang K.C., Helms J.A., Chang H.Y. (2009). Regeneration, repair and remembering identity: The three Rs of *HOX* gene expression. Trends Cell Biol.

[77] Yamaguchi Y., Itami S., Tarutani M., Hosokawa K., Miura H., Yoshikawa K. (1999). Regulation of keratin 9 in nonpalmoplantar keratinocytes by palmoplantar fibroblasts through epithelial-mesenchymal interactions. J. Investig. Dermatol.

[78] Yamaguchi Y., Itami S., Watabe H., Yasumoto K., Abdel-Malek Z.A., Kubo T., Rouzaud F., Tanemura A., Yoshikawa K., Hearing V.J. (2004). Mesenchymal-epithelial interactions in the skin: Increased expression of dickkopf1 by palmoplantar fibroblasts inhibits melanocyte growth and differentiation. J. Cell Biol.

[79] Yamaguchi Y., Passeron T., Hoashi T., Watabe H., Rouzaud F., Yasumoto K., Hara T., Tohyama C., Katayama I., Miki T. (2008). Dickkopf 1 (DKK1) regulates skin pigmentation and thickness by affecting Wnt/β-catenin signaling in keratinocytes. FASEB J.

[80] Boulton A.J., Kirsner R.S., Vileikyte L. (2004). Clinical practice. Neuropathic diabetic foot ulcers. N. Engl. J. Med.

[81] Brem H., Kirsner R.S., Falanga V. (2004). Protocol for the successful treatment of venous ulcers. Am. J. Surg.

[82] Charles C.A., Tomic-Canic M., Vincek V., Nassiri M., Stojadinovic O., Eaglstein W.H., Kirsner R.S. (2008). A gene signature of nonhealing venous ulcers: Potential diagnostic markers. J. Am. Acad. Dermatol.

[83] Zaulyanov L., Kirsner R.S. (2007). A review of a bi-layered living cell treatment (Apligraf) in the treatment of venous leg ulcers and diabetic foot ulcers. Clin. Interv. Aging.

[84] Schmidt C. (2011). FDA approves first cell therapy for wrinkle-free visage. Nat. Biotechnol.

[85] Yang N.B., Garza L.A., Foote C.E., Kang S., Meyerle J.H. (2012). High prevalence of stump dermatoses 38 years or more after amputation. Arch. Dermatol.

[86] Harper R.A., Grove G. (1979). Human skin fibroblasts derived from papillary and reticular dermis: Differences in growth potential *in vitro*. Science.

[87] Mine S., Fortunel N.O., Pageon H., Asselineau D. (2008). Aging alters functionally human dermal papillary fibroblasts but not reticular fibroblasts: A new view of skin morphogenesis and aging. PLoS One.

[88] Schonherr E., Beavan L.A., Hausser H., Kresse H., Culp L.A. (1993). Differences in decorin expression by papillary and reticular fibroblasts *in vivo* and *in vitro*. Biochem. J..

[89] Janson D.G., Saintigny G., van Adrichem A., Mahe C., El Ghalbzouri A. (2012). Different gene expression patterns in human papillary and reticular fibroblasts. J. Investig. Dermatol.

[90] Pageon H., Zucchi H., Asselineau D. (2012). Distinct and complementary roles of papillary and reticular fibroblasts in skin morphogenesis and homeostasis. Eur. J. Dermatol.

[91] Tajima S., Pinnell S.R. (1981). Collagen synthesis by human skin fibroblasts in culture: Studies of fibroblasts explanted from papillary and reticular dermis. J. Investig. Dermatol.

[92] Sorrell J.M., Baber M.A., Caplan A.I. (2004). Site-matched papillary and reticular human dermal fibroblasts differ in their release of specific growth factors/cytokines and in their interaction with keratinocytes. J. Cell. Physiol.

[93] Varkey M., Ding J., Tredget E.E. (2011). Differential collagen-glycosaminoglycan matrix remodeling by superficial and deep dermal fibroblasts: Potential therapeutic targets for hypertrophic scar. Biomaterials.

[94] Jahoda C.A., Reynolds A.J. (2001). Hair follicle dermal sheath cells: Unsung participants in wound healing. Lancet.

[95] Botchkarev V.A., Paus R. (2003). Molecular biology of hair morphogenesis: Development and cycling. J. Exp. Zool. B.

[96] Kramann R., DiRocco D.P., Humphreys B.D. (2013). Understanding the origin, activation and regulation of matrix-producing myofibroblasts for treatment of fibrotic disease. J. Pathol.

[97] Qiao J., Zawadzka A., Philips E., Turetsky A., Batchelor S., Peacock J., Durrant S., Garlick D., Kemp P., Teumer J. (2009). Hair follicle neogenesis induced by cultured human scalp dermal papilla cells. Regen. Med.

[98] Hardy M.H., van Exan R.J., Sonstegard K.S., Sweeny P.R. (1983). Basal lamina changes during tissue interactions in hair follicles–An *in vitro* study of normal dermal papillae and vitamin A-induced glandular morphogenesis. J. Investig. Dermatol.

[99] Ramos R., Guerrero-Juarez C.F., Plikus M.V. (2013). Hair follicle signaling networks: A dermal papilla-centric approach. J. Investig. Dermatol.

[100] Chen C.C., Chuong C.M. (2012). Multi-layered environmental regulation on the homeostasis of stem cells: The saga of hair growth and alopecia. J. Dermatol. Sci.

[101] Yang C.C., Cotsarelis G. (2010). Review of hair follicle dermal cells. J. Dermatol. Sci.

[102] Christoph T., Muller-Rover S., Audring H., Tobin D.J., Hermes B., Cotsarelis G., Ruckert R., Paus R. (2000). The human hair follicle immune system: Cellular composition and immune privilege. Br. J. Dermatol.

[103] Hill R.P., Haycock J.W., Jahoda C.A. (2012). Human hair follicle dermal cells and skin fibroblasts show differential activation of NFκB in response to pro-inflammatory challenge. Exp. Dermatol.

[104] Reynolds A.J., Lawrence C., Cserhalmi-Friedman P.B., Christiano A.M., Jahoda C.A. (1999). Trans-gender induction of hair follicles. Nature.

[105] McElwee K.J., Kissling S., Wenzel E., Huth A., Hoffmann R. (2003). Cultured peribulbar dermal sheath cells can induce hair follicle development and contribute to the dermal sheath and dermal papilla. J. Invest. Dermatol.

[106] Zhang X., Deng Z., Wang H., Yang Z., Guo W., Li Y., Ma D., Yu C., Zhang Y., Jin Y. (2009). Expansion and delivery of human fibroblasts on micronized acellular dermal matrix for skin regeneration. Biomaterials.

[107] Higgins C.A., Chen J.C., Cerise J.E., Jahoda C.A., Christiano A.M. (2013). Microenvironmental reprogramming by three-dimensional culture enables dermal papilla cells to induce *de novo* human hair-follicle growth. Proc. Natl. Acad. Sci. USA.

[108] Higgins C.A., Richardson G.D., Ferdinando D., Westgate G.E., Jahoda C.A. (2010). Modelling the hair follicle dermal papilla using spheroid cell cultures. Exp. Dermatol.

[109] Morimoto N., Takemoto S., Kanda N., Ayvazyan A., Taira M.T., Suzuki S. (2011). The utilization of animal product-free media and autologous serum in an autologous dermal substitute culture. J. Surg. Res.

[110] Mazlyzam A.L., Aminuddin B.S., Saim L., Ruszymah B.H. (2008). Human serum is an advantageous supplement for human dermal fibroblast expansion: Clinical implications for tissue engineering of skin. Arch. Med. Res.

[111] Aoi N., Inoue K., Chikanishi T., Fujiki R., Yamamoto H., Kato H., Eto H., Doi K., Itami S., Kato S. (2012). 1α,25-Dihydroxyvitamin D3 modulates the hair-inductive capacity of dermal papilla cells: Therapeutic potential for hair regeneration. Stem Cells Transl. Med.

[112] Lee S.H., Yoon J., Shin S.H., Zahoor M., Kim H.J., Park P.J., Park W.S., Min do S., Kim H.Y., Choi K.Y. (2012). Valproic acid induces hair regeneration in murine model and activates alkaline phosphatase activity in human dermal papilla cells. PLoS One.

[113] Yamauchi K., Kurosaka A. (2009). Inhibition of glycogen synthase kinase-3 enhances the expression of alkaline phosphatase and insulin-like growth factor-1 in human primary dermal papilla cell culture and maintains mouse hair bulbs in organ culture. Arch. Dermatol. Res.

[114] Zheng Y., Du X., Wang W., Boucher M., Parimoo S., Stenn K. (2005). Organogenesis from dissociated cells: Generation of mature cycling hair follicles from skin-derived cells. J. Investig. Dermatol.

[115] Kang B.M., Kwack M.H., Kim M.K., Kim J.C., Sung Y.K. (2012). Sphere formation increases the ability of cultured human dermal papilla cells to induce hair follicles from mouse epidermal cells in a reconstitution assay. J. Investig. Dermatol.

[116] Thangapazham R.L., Klover P., Wang J.A., Zheng Y., Devine A., Li S., Sperling L., Cotsarelis G., Darling T.N. (2014). Dissociated human dermal papilla cells induce hair follicle neogenesis in grafted dermal-epidermal composites. J. Investig. Dermatol.

[117] McElwee K., Hall P.D.D., Hoffmann R. (2013). Towards a cell-based treatment for androgenetic alopecia in men and women: 12-Month interim safety results of a phase 1/2a clinical trial using autologous dermal sheath cup cell injections.

